# Locating Femoral Vein by Anatomic Landmarks: A Cadaveric Study

**DOI:** 10.7759/cureus.81267

**Published:** 2025-03-27

**Authors:** Anasuya Ghosh, Satabdi Sarkar, Yashu Bhardwaj, Biswabina Ray, Anirban Dasgupta

**Affiliations:** 1 Anatomy, All India Institute of Medical Sciences, Kalyani, Kalyani, IND; 2 Anatomy, University College of Medical Sciences, University of Delhi, New Delhi, IND

**Keywords:** anterior superior iliac spine, cadaveric lower limbs, depth from skin surface, femoral vein, pubic symphysis

## Abstract

Background: In circumstances of emergency or challenging peripheral access, the femoral vein serves as a vital intravenous access channel. The vein is commonly located by palpating the femoral arterial pulse inferior to the mid-inguinal point or by the ‘V’ technique. As femoral arterial pulse may not be non-palpable in some cases, some distances from nearby anatomic landmarks might help to locate the femoral vein for cannulation.

Materials and methods: In 54 dissected cadaveric lower limbs, the distances of the femoral vein from the anterior superior iliac spine, the symphysis pubis, and the skin surface were measured to prepare a dataset for locating the vein with the help of these data. The values were statistically analyzed.

Result: The mean distance of the femoral vein from the anterior superior iliac spine was 80.16±8.96 mm, the mean distance from the symphysis pubis was 66.77±11.08 mm, and the mean depth of the femoral vein from the skin surface was 20.93±8.84 mm. All the distances and skin depths were higher in female limbs; however, only the depth from the skin surface was statistically significant across the genders.

Conclusion: These datasets might be useful as additional support while performing femoral vein cannulation in complicated and challenging cases where the facility of radiological monitoring is not available.

## Introduction

In circumstances of emergency or challenging peripheral access, the femoral vein serves as a vital intravenous access channel. Extensive intravenous access can be obtained with femoral vein cannulation, which is comparatively simple. Traditionally, the femoral artery pulsation next to it can be felt to determine its identity [1]. 

The major deep vein in the lower limb is the femoral vein. The femoral vein is the continuation of the popliteal vein proximal to the adductor hiatus at the front of the thigh and femoral triangle. It continues as the external iliac vein while entering the pelvis beneath the inguinal ligament [2]. At the level of the inguinal ligament, the hip joint and psoas muscle are situated precisely beneath the femoral neurovascular bundle. The femoral vein is located medial to the femoral artery within the femoral triangle and is a content of the femoral sheath. The femoral vein commonly has two superficial tributaries: the great saphenous and anterior saphenous veins. During cannulation, it is possible for these veins to become accidentally cannulated and initially misdiagnosed as femoral veins [3].

The most commonly used landmark to locate the femoral vein for cannulation is the femoral artery pulse, which is located halfway between the anterior superior iliac spine (ASIS) and pubic symphysis [4]. Some people, however, have a femoral artery pulse that is too feeble to detect [1]. The important reference point for locating the femoral artery is the mid-inguinal point, which approximates the point where the external iliac artery continues under the inguinal ligament as the femoral artery. This mid-inguinal point can be utilized during cardiac arrest or in the absence of a palpable femoral pulse to locate the femoral vein, as the femoral vein lies just medial to the artery at this position [4]. An essential procedural landmark, the inguinal or groin crease, approximates the inguinal ligament. It is strategically advised to target the most superficial point of the femoral vein below the inguinal crease to prevent problems identifying and compressing bleeding difficulties related to the iliac vein or artery puncture [1]. 

Hence, it is necessary to know the exact location of the femoral vein with respect to the surrounding anatomical landmarks in addition to the femoral pulsation. The present study has been conducted to identify the exact location of the femoral vein in relation to nearby bony landmarks and skin surface since very little research has been done on these particular parameters. The primary objective of the study was to prepare a dataset by measuring the distances of the femoral vein from the nearby bony landmarks and the skin surface in cadavers to locate the vein at the inguinal crease easily.

## Materials and methods

This cross-sectional study was conducted on 54 cadaveric lower limbs at the Wet Cadaveric Lab at the Department of Anatomy of the All India Institute of Medical Sciences (AIIMS), Kalyani, West Bengal, India, from July 2022 to May 2024. The study was approved by the Institutional Ethics Committee of AIIMS, Kalyani (Ref. No. IEC/AIIMS/Kalyani/Meeting/2022/13). 

Of the cadaveric lower limbs, 36 were from male cadavers and 18 from female. The convenience sampling method was used for this study, and cadavers (aged between 58 and 82 years) used for undergraduate teaching were utilized. Limbs with a damaged groin area, femoral artery embalming, a history of vascular or pelvic surgery, and congenital or acquired vascular abnormalities were excluded from the study.

**Figure 1:**
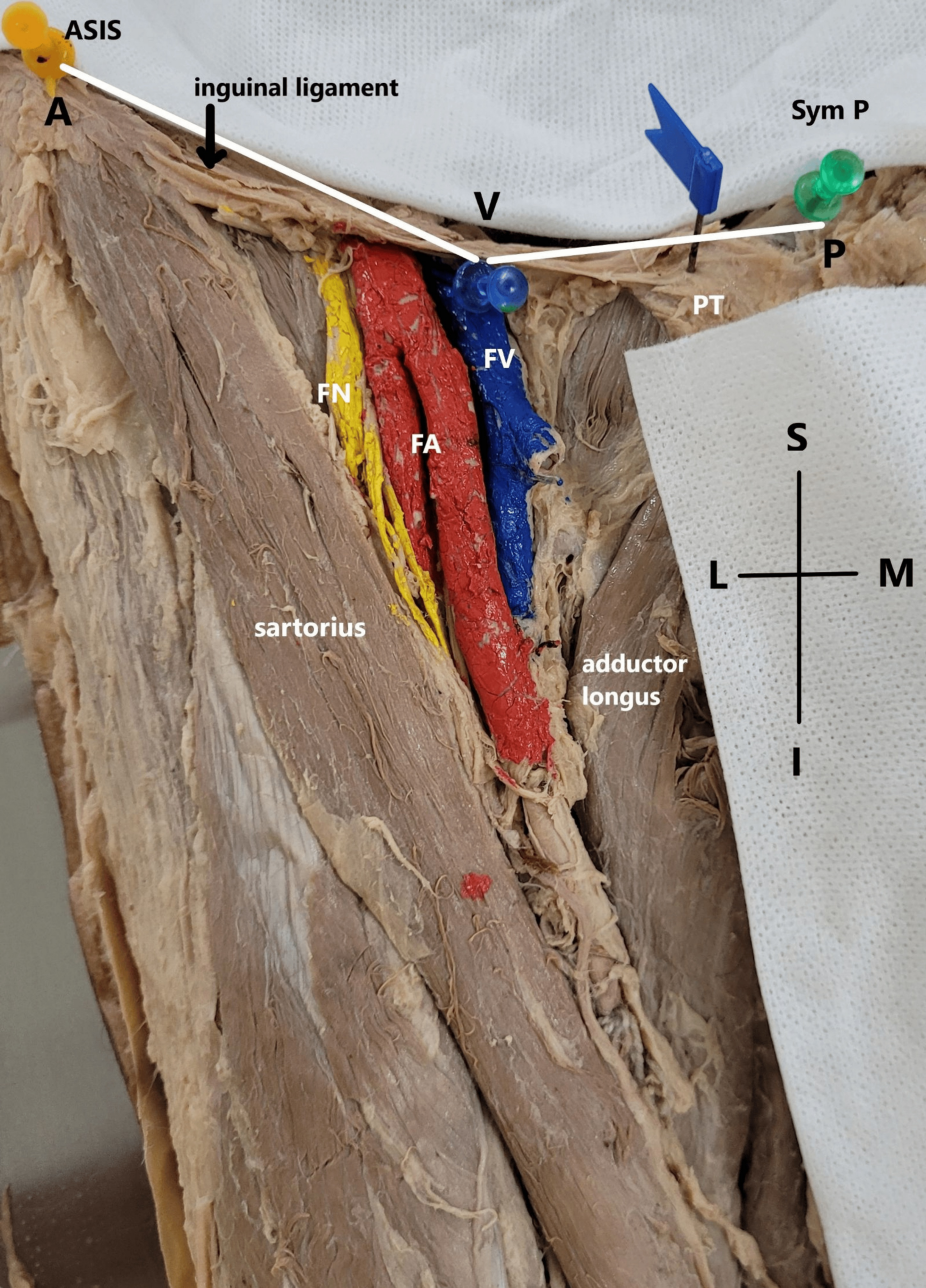
Distances between the femoral vein and nearby bony landmarks in a dissected cadaver ASIS: anterior superior iliac spine; SymP: symphysis pubis; PT: pubic tubercle; FV: femoral vein; FA: femoral artery; FN: femoral nerve; S: superior; I: inferior; L: lateral; M: medial

The distances from nearby fixed bony landmarks were measured to establish a data set to locate the femoral vein accurately in the following manner as depicted in Figure 1: (a) The horizontal distance from the mid-point of the femoral vein at the inguinal crease to the pubic symphysis (SymP), (b) The oblique distance from the mid-point of the femoral vein at the inguinal crease to the mid-point of the ASIS along the crease, and (c) The depth of the femoral vein from the skin surface at the inguinal crease.

The cadaveric limbs were dissected following the standard protocol; after the removal of skin and careful removal of superficial and deep fascia, the boundaries and contents of the femoral triangle were exposed. The bony landmarks, i.e., ASIS, SymP, pubic tubercle (PT), and midpoint of FV, were marked for the measurement. The measurements were done by digital calipers and non-stretchable measuring tape. All the measurements were taken while keeping the cadavers in a supine position with fully extended lower limbs. Two fixed investigators took the same measurements, and the average value was considered. The accuracy of the caliper was monitored through regular calibration.

All the data were recorded and analyzed using Microsoft Excel (Microsoft Corporation, Redmond, Washington, United States) and GraphPad Prism (Dotmatics, Boston, Massachusetts, United States). P values < 0.05 were regarded as statistically significant.

## Results

The mean distance between the femoral vein and ASIS was 80.16±8.96 mm, and between the femoral vein and SymP, it was 66.77±11.08 mm, considering all 54 cadaveric limbs (Table 1). There was no statistically significant difference across the right and left sides of the same gender.

**Table 1:** Comparison between distances from bony landmarks ASIS: anterior superior iliac spine; SymP: symphysis pubis; MPFV: mid-point of femoral vein

Distances	Male			Female			Total (n=54)
	Right side (n=20)	Left side (n=16)	P value	Right side (n=9)	Left side (n=9)	P value	
ASIS to MPFV	79.11±10.34 mm	80.21±9.81 mm	0.74	79.48±6.23 mm	83.06±6.77 mm	0.26	80.16±8.96 mm
SymP to MPFV	65.99±9.2 mm	64.26±9.49 mm	0.58	72.8±16.001 mm	66.98±11.59 mm	0.38	66.77±11.08 mm

The female limbs had higher mean values for both distances than the male limbs, but the difference was not statistically significant (Table 2).

**Table 2:** Comparison between two distances across genders ASIS: anterior superior iliac spine; SymP: symphysis pubis; MPFV: mid-point of femoral vein

Variables	Mean±SD (mm)	Range (mm)	t value	P value
ASIS to MPFV in male (n=36)	79.597±9.982	56-98.4	0.65	0.5211 (No statistical significance)
ASIS to MPFV in female (n=18)	81.27±6.576	69.9-91.2		
SymP to MPFV in male (n=36)	65.21±9.240	48-82.5	1.48	0.1461 (No statistical significance)
SymP to MPFV in female (n=18)	69.88±13.853	42.6-87.9		

The mean depth of the femoral vein from the skin surface was 20.93±8.84 mm at the inguinal crease. The female limbs had significantly higher values (Table 3).

**Table 3:** Distance of superficial surface of skin and femoral vein

Depth of femoral vein from the superficial surface of skin	Mean (mm)	Standard Deviation (mm)	Range (mm)	Mean across gender (n=54)	t value	p value
Male (n=36)	19.15	9.067	6.1-37.5	20.93± 8.84	2.17	0.034 (statistically significant)
Female (n=18)	24.50	7.35	11- 33.7			

In 100% of the cases, some degree of overlapping was observed in femoral vessels; the vein was located posteromedial to the artery and was marginally overlapped by the artery. Proper dissection of the femoral sheath was required to expose them separately.

## Discussion

In the present study, the distance of the femoral vein at the inguinal crease has been measured from the ASIS and midpoint of the SymP in 54 cadaveric lower limbs (36 male, 18 female). Both distances were higher in female limbs than in male limbs. However, the difference was not statistically significant. The higher distances in females could be due to a wider pelvis in them. The depth of the vein at the inguinal crease from the skin surface was also measured in both genders, where the depth was significantly higher in females. A dataset of the above parameters has been prepared to assist in locating the femoral vein more accurately using the bony landmarks. This study was conducted to accurately identify the femoral vein for cannulation and other clinical purposes using the nearby bony landmarks. The data collected to locate the femoral vein in the present study is quite rare in the literature.

In the present study, the location of the femoral vein from nearby palpable bony landmarks was assessed at the level of the inguinal crease. In a study by Canteras et al., the inguinal crease has been recommended for transverse incision to access this area easily for surgical procedures and better postoperative healing [5].

In the present study, approximately 0.5-2.3 mm overlapping of the femoral vein and artery was observed across all genders at the level of the inguinal crease. Based on this observation, it is suggested to insert the needle medial to the mid-point of the femoral vein while putting the cannula to avoid any inadvertent injury to the artery. A similar observation has been recorded in 95% of cases in an ultrasonographic study of femoral vessels in an emergency setup [6].

Bannon et al. emphasized the importance of surface anatomic landmarks for central venous cannulation to be successful. They mentioned that surface landmarks remain crucial to safe central venous cannulation, even though ultrasound (US) adds a layer of orientation [7].

Pietroboni et al. studied the landmark method (LM) versus US-guided insertion of femoral venous catheters in the pediatric intensive care unit. The cannulation success was found on the first try, and overall success was statistically significantly greater in the US group than the LM. The US group had a decreased rate of femoral artery punctures compared to LM [8].

Some clinical researchers have mentioned the ‘V technique’ as an effective landmark technique to locate the femoral vein for cannulation [2,7]. In the V technique, the thumb is placed lateral to the pubic tubercle and the index finger is placed over the ASIS, and the femoral vein is supposed to be cannulated through the base of the V shape created by the first web space of the hand. Utilizing the landmark technique by palpating the ASIS, SymP, and femoral arterial pulsation, femoral venous cannulation can be done. Still, the US-guided method has been recommended for a higher success rate with the least number of attempts. Tantri et al. studied a comparison of the V technique and femoral arterial palpation technique’s accuracy in identifying the femoral vein’s cannulation site [1]. They found that the accuracy of the femoral artery pulsation technique was 96.5%, while the accuracy of the V technique was 93.9%. However, the accuracy of the two approaches did not have a statistically significant difference, according to the McNemar analysis (p = 0.549). The skin projection of the femoral vein midpoint and the distance of the femoral vein cannulation location predicted by both methods showed a statistically significant positive association (r = 0.548; p < 0.001) [1].

It was observed that in hypotensive patients, the femoral arterial pulsation might not be palpable [9]. Interestingly, in a study by Seyahi et al., 114 patients with femoral venous cannulation were studied, and non-palpable femoral arteries were found in 14% of patients [10]. It indicates that the femoral arterial pulsation, the most dependable palpable landmark to locate the femoral vein, may not apply to some people. The parameters of the present study could help locate the femoral vein along with the V technique to establish a central line utilizing the femoral vein at remote healthcare facilities. Saguel et al. suggested that the most effective way to achieve the best skill level for central venous catheter (CVC) placement is to combine knowledge from anatomic landmark techniques and US-guided CVC placement [11].

In a US study on living patients by Zambetti et al., the mean depth of FV was reported as 18 mm, and depth was correlated with the weight of the person [12]. In another study by Seyahi et al., the mean depth was 20.7±6.5 vs 14.6±5.1 mm in overweight versus normal body weight patients, and the depth of femoral vessels was correlated with the body weight of the person [10].

In the present study, the mean depth of the femoral vein was 19.15±9.07 mm in male cadavers and 24.50±7.35 mm in female cadavers. The higher depth in females could be due to more subcutaneous fat in the lower abdomen, as women have relatively more adipose tissue in the hips and thighs [13]. The findings were comparable to previous studies.

The measurement of distances of the femoral vein from nearby bony landmarks has rarely been done before, making the present study novel.

There were certain limitations in the present study. The height, weight, and body mass index of the cadavers were not recorded. The small sample size and nonrepresentative samples could minimize the chance of generalization of the study results beyond the sample. Future studies should be conducted on a larger sample size with a collection of other relevant data. Similar studies using radiologic modalities could be planned on living patients too.

## Conclusions

In the present study, it was found that the mean distance of the femoral vein from the anterior superior iliac spine was 80.16±8.96 mm, the mean distance from the symphysis pubis was 66.77±11.08 mm, and the mean depth of the femoral vein from the skin surface was 20.93±8.84 mm. This reference data set might be used as an additional backup while locating the femoral vein for clinical intervention by landmark technique.
